# Oral infection of mice with *Salmonella enterica *serovar Typhimurium causes meningitis and infection of the brain

**DOI:** 10.1186/1471-2334-7-65

**Published:** 2007-06-27

**Authors:** Mark E Wickham, Nat F Brown, John Provias, B Brett Finlay, Brian K Coombes

**Affiliations:** 1 Michael Smith Laboratories, University of British Columbia, Vancouver, BC., Canada; 2 Department of Neuropathology, Hamilton Health Sciences Corporation, Hamilton, ON., Canada; 3 Department of Biochemistry and Biomedical Sciences, McMaster University, Hamilton, ON, Canada, and the Laboratory for Foodborne Zoonoses, Public Health Agency of Canada, Guelph, ON., Canada; 4 Phillips Ormonde Fitzpatrick, Level 21, 367 Collins Street, Melbourne 3000, Australia; 5 Institute for Glycomics, Griffith University-Gold Coast Campus, PMB 50, Gold Coast Mail Centre, Gold Coast, Queensland 9726, Australia

## Abstract

**Background:**

*Salmonella *meningitis is a rare and serious infection of the central nervous system following acute *Salmonella enterica *sepsis. For this pathogen, no appropriate model has been reported in which to examine infection kinetics and natural dissemination to the brain.

**Methods:**

Five mouse lines including C57BL/6, Balb/c, 129S6-*Slc11a1tm1*^*Mcg*^, 129S1/SvImJ, B6.129-*Inpp5dtm1*^*Rkh *^were used in the murine typhoid model to examine the dissemination of systemic *Salmonella enterica *serovar Typhimurium following oral infection.

**Results:**

We report data on spontaneous meningitis and brain infection following oral infection of mice with *Salmonella enterica *serovar Typhimurium.

**Conclusion:**

This model may provide a system in which dissemination of bacteria through the central nervous system and the influence of host and bacterial genetics can be queried.

## Background

*Salmonella *species are Gram-negative, facultative intracellular bacteria that are distributed globally. Two recognized species of *Salmonella *include *S. enterica *and *S. bongori*, with *S. enterica *serovars Typhimurium, Typhi and Enteriditis causing the vast majority of human infections worldwide. Humans are infected with *S. enterica *though contaminated food and water and present with a range of acute symptoms including gastroenteritis, fever, and headache. Although systemic infections with *S*. Typhi are uncommon in developed countries, typhoid remains a significant public health problem in the developing world [1]. Infections with non-typhoidal strains of *Salmonella *are a global burden, with an estimated 1.4 million cases in the United States alone [2].

*Salmonella *meningitis is an uncommon complication of salmonellosis, occurring more frequently in neonates and infants [3,4], although adult cases are reported. While considered rare in the developed world, *Salmonella *is a common cause of enterobacterial meningitis in Africa, Brazil and Thailand [4,5]. Cases in adults of *Salmonella *infection report colonization of the cerebrospinal fluid, fatal brain abscesses caused by intracranial colonization of *S. enterica *serotype Typhimurium [6], adult *Salmonella *meningitis [7] and CSF pleocytosis [7]. Mortality rates are typically high, especially in infants where rates have been 60% [8]. Other major issues concerning *Salmonella *meningitis is a high treatment failure rate, high relapse rate and considerable neurological sequelae in those that survive, including mental retardation, cerebral palsy, and visual and hearing impairment [3,4,8-11]. The treatment of such complicated cases is made more difficult by the lack of a priori knowledge of the pathogen involved, restrictions in pediatric use of certain antibiotics, the sharp and continued rise in antimicrobial resistance and the proliferation of multi-drug resistant organisms in community settings. The 1970's to early 1990's witnessed the emergence of *Salmonella *isolates resistant to the front-line antimicrobial chloramphenicol, as well as cotrimoxazole, ampicillin and amoxicillin [12]. In more contemporary medicine, multi drug-resistant *Salmonella *such as *S*. Typhimurium strain DT104 is a constant reminder that new anti-infectives against novel targets are in demand. DT104 is commonly resistant to ampicillin, chloramphenicol, florfenicol, streptomycin, spectinomycin, sulfonamides and tetracycline [13] and infections with multi-drug resistant *S. enterica *isolates are associated with higher mortality rates than infection with susceptible strains [14]. This apparent link between drug resistance and virulence is not well understood but deserves considerable attention.

The mechanisms of how *Salmonella *gains access to the CNS and brain are not known. Outstanding questions include (i) how does *Salmonella *disseminate from the intestinal mucosa to the CNS? (ii) how do bacteria gain access to the brain? (iii) what cell types are utilized for transport to the brain and intracellular replication within this compartment? (iv) what role does host genetics and immune repertoire play in this process? Lack of an appropriate animal model has made it difficult to address these questions. An animal model in rabbits of experimental *S. enterica *serotype Enteriditis meningitis has been reported [15], although this model involved direct inoculation of the cerebral spinal fluid using an intracisternally placed needle. Currently there is a dearth of reports that describe the natural progression of salmonellosis from the intestinal mucosa, though the CNS and to the brain. Here we report that the widely used oral infection model of salmonellosis fulfills the criteria of natural dissemination though a susceptible host animal from the intestinal mucosa to the brain. This is accompanied by meningitis and a reproducible behavioral manifestation of intracranial infection that occurs in at least five genetically distinct mouse lines and that correlates significantly with the bacterial load in the brain. The development of animal models of bacterial meningitis and intracranial infections will permit a better understanding of the mechanisms of bacterial dissemination, the kinetics of natural disease progression, and the influence of host genetics on this process. Such models will also be necessary for testing investigational antimicrobials and vaccines for therapeutic value against bacterial meningitis.

## Methods

### Bacteria and culture conditions

*Salmonella enterica *serovar Typhimurium strain SL1344 [16] was used throughout this study. Bacteria were routinely cultured in LB broth and on solid LB agar plates containing streptomycin at 50 μg ml^-1^. Prior to animal infections, bacteria were cultured overnight in LB broth, washed in a buffer containing 0.1 M HEPES (pH 8.0) and 0.9% sodium chloride, and resuspended in the same buffer to ~10^7 ^– 10^9 ^colony forming units (cfu) per ml.

### Experimental animals

Experimental animals were female mice between 8 weeks to 12 weeks of age. Five different mouse strains were used in these experiments and include: C57BL/6 (Jackson Laboratories), Balb/c (Jackson Laboratories), 129S6-*Slc11a1tm1*^*Mcg *^(formerly 129/sv *Ity/Nramp*^-/-^) [17], 129S1/SvImJ (Jackson Laboratories), B6.129-*Inpp5dtm1*^*Rkh *^(formerly 129sv/*SHIP-1*^-/- ^F1) [18]. Animals were obtained with full health reports, were deemed to be healthy and free of infection and were housed under specific pathogen free conditions.

### Animal infections and tissue samples

All animal experiments were conducted as approved by the local Animal Ethics Board and pursuant to guidelines set out by the Canadian Council on Animal Care. Animal infections were carried out by inoculating mice per os using a gavage needle with approximately 10^6 ^to 10^8 ^wild type *S. enterica *serotype Typhimurium strain SL1344 in a 0.1 ml volume. Infected animals were examined twice daily for signs of terminal morbidity and animals that had become moribund or that exhibited a balance defect were euthanised by cervical dislocation. At necropsy the brain was removed, dissected into right and left hemispheres with one hemisphere processed for pathology and the other placed in 1 ml of sterile phosphate buffered saline. This latter sample was homogenized in a tissue homogenizer (Polytron, Kinematica) and the homogenate was diluted in sterile PBS and plated on solid LB agar containing streptomycin to enumerate *S*. Typhimurium cfu. Bacterial load was expressed as cfu/organ.

### Pathology scoring

For pathologic scoring, brain tissues were fixed in 2% paraformaldehyde in routine fashion [19] and sectioned for histologic examination. Each brain was processed in one paraffin block and three sections were cut and stained with hematoxylin and eosin (H/E), Gram, and napthol esterase to identify mast cells. A neuropathologist examined all brain sections blindly. The degree of inflammation was scored on a 0 to 4+ scale, 0 representing no inflammation and 4+ representing involvement of the entire contour of the sub-arachnoid space of the section.

## Results

### Observations on infected mice

**Figure 1:**
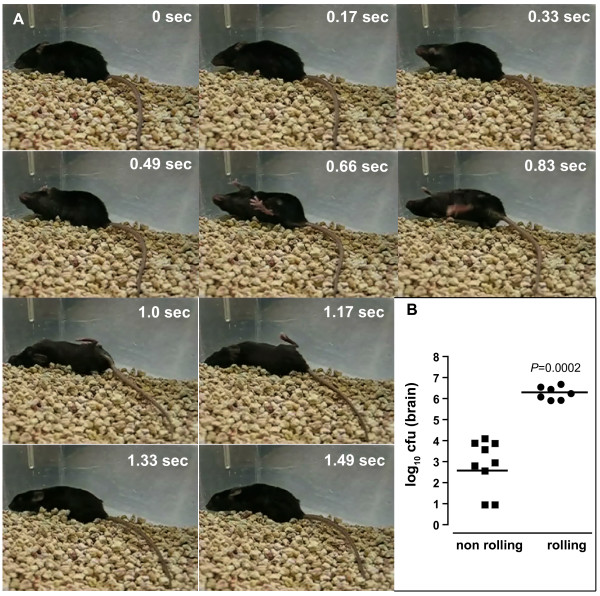
**Rolling behavior of *Salmonella *infected mice**. (A). Still frames of a C57BL/6 mouse infected with *Salmonella enterica *serovar Typhimurium (plus Additional file 1) which shows rolling behavior as a prominent feature of intracranial infection. Rotation speeds of 1 rotation per 0.3 seconds to 1.5 seconds were observed. Rotary motion was observed in either direction, though only unidirectional motion was observed for any given mouse. (B). Bacterial load in the brains of rolling and non-rolling C57BL/6 mice. Infected mice were sacrificed and the brain removed as described in the text. Brain homogenates were plated on solid LB microbiological agar for enumeration of bacterial colony forming units (cfu). Each data point represents one animal. Shown is the data scatter of log-transformed cfu per organ from each animal, with the horizontal line representing the geometric mean (*P *= 0.0002, Mann Whitney).

Our studies on *Salmonella enterica *pathogenesis involve infection of the mouse in order to examine bacterial virulence factors that are essential during discrete stages of infection. During the acute phase of infection, mice develop overt signs of infection (hunched posture, reduced movement, loss of body weight, piloerection). During the course of these studies over several years, we became interested in a proportion of mice that developed a neurological abnormality with balance defect following infection. These mice exhibited an exaggerated lean to one side of the body and longitudinal spinning and rotary motion as the most prominent manifestation of infection. Rolling occurred in either direction (however only unidirectional movement was observed for any given mouse) ranging from intermittent rolling through to constant rolling (Figure 1, and Additional file 1). Neurological deficits were not unique to one particular strain of mouse, as we observed this behaviour in five distinct mouse lines including wild type C57BL/6, wild type BALB/c, 129S1/SvImJ, 129S6-*Slc11a1tm1*^*Mcg*^, and B6.129-*Inpp5dtm1*^*Rkh*^.

### Examination of bacterial load in infected mice

The observed neurological deficits following infection lead us to speculate that an intracranial infection with *Salmonella *was taking place in affected mice. To examine the dissemination of *S*. Typhimurium in mice exhibiting rolling behavior, bacterial load was examined in both rolling and non-rolling infected mice. Brains of mice exhibiting neurological deficit had significantly higher bacterial loads (mean, 2.66 ± 0.66 × 10^6 ^cfu/organ) than infected mice without overt signs of neurological deficit (mean, 4.21 ± 1.72 × 10^3 ^cfu/organ) (*P *= 0.0002, Mann Whitney) (Figure 1). Whereas some non-rolling mice had no detectable cfu in the brain (approximately 10–20% of animals), this was never the case for infected mice that had developed neurological deficits.

### Pathologic examination of mouse brains

**Figure 2:**
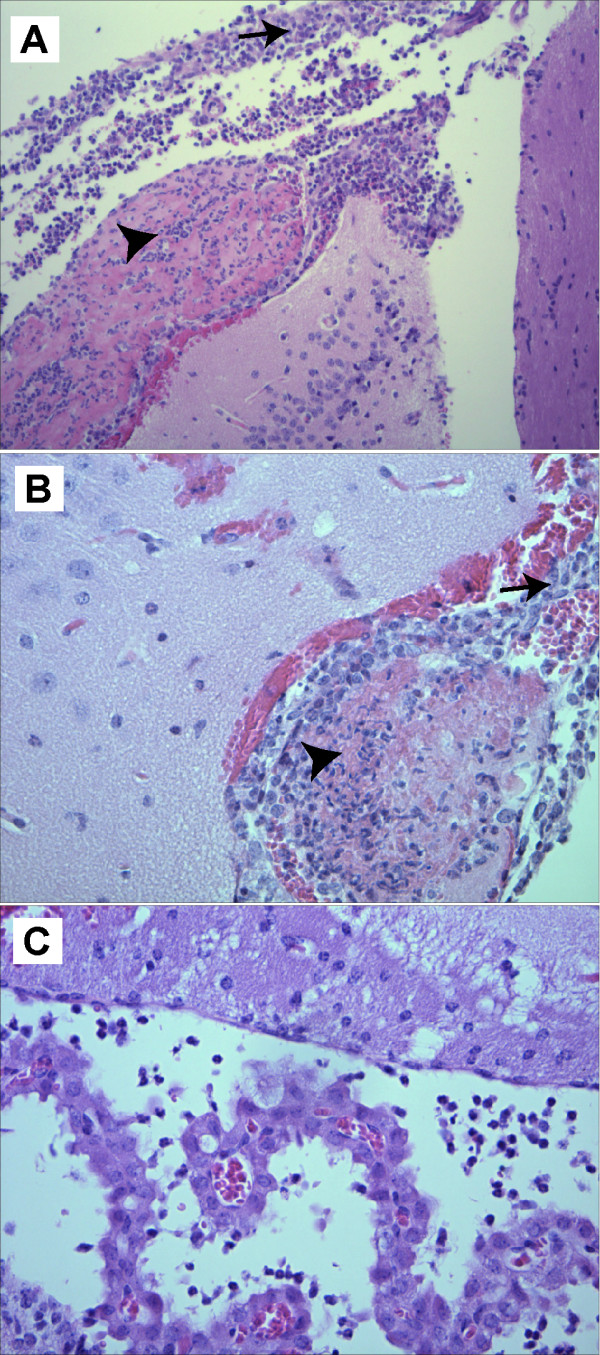
**Histopathology of infected brain sections**. (A, B) Coronal sections of brain with sub-arachnoid meningeal inflammation (arrowheads). Macrophages are the predominant cellular infiltrate in these fields. Thrombosed sub-arachnoid vessels are marked with arrows. H&E; mag, 100×. (C) Area of ventriculitis seen in the brain of mouse #6. H&E; mag, 200×.

The finding that a proportion of mice developed neurological deficits following oral infection with *S*. Typhimurium and that these mice had a significantly greater bacterial load in the brain compared to non-affected mice lead us to test whether mice developed meningitis following infection. Mouse brains that were processed for histopathology were randomized and coded and scored by a neuropathologist who was blinded to the treatment history and clinical outcome of the mouse. The principal clinical finding was the presence of patchy meningitis confined to the sub-arachnoid space in all mice with neurological deficit (see Table 1). The meningitis was composed of a mixture of acute and chronic inflammatory cells, chiefly neurophils and macrophages. In general the meningitis was consistent with a sub-acute time frame reflecting a process of a few days to one week in duration. The inflammation was non-necrotizing and no granulomas were present. The degree of inflammation was scored on a 0 to 4+ scale, 0 representing no inflammation and 4+ representing involvement of the entire contour of the sub-arachnoid space of the section (data summarized in Table 1). Some of the brains had thrombosed sub-arachnoid vessels in areas of meningitis, however none of the cases had any cerebral infarction or encephalitis. One of the cases with more intense meningitis also had a ventriculitis (Figure 2). There was no obvious correlation between development of meningitis and bacterial load in other systemic sites of infection including the liver and spleen. Bacterial load in these sites is typically variable in infected mice and ranged from ~1 × 10^4 ^to 5 × 10^6 ^cfu per organ (data not shown) regardless of the clinical diagnosis of the animal.

**Table 1:** Clinical Summary

Mouse	Treatment	Behaviour	Clinical Features ^*a*^	Meningitis score	Genetic background
1	uninfected	non-rolling	normal	0	C57BL/6
2	infected	non-rolling	normal	0	C57BL/6
3	infected	rolling	meningitis, thrombosis	1+	Balb/c
4^*b*^	infected	rolling	meningitis	2+	C57BL/6
5	infected	rolling	meningitis	0.5+	Balb/c
6	infected	rolling	meningitis, thrombosis, ventriculitis	2+	Balb/c
7	infected	rolling	meningitis	1+	129S6-*Slc11a1tm1*^*Mcg*^

^*a *^slides were scored with the observer blinded to mouse history

^*b *^mouse shown in Additional file 1

## Discussion

The occurrence of meningitis caused by serovars of *Salmonella enterica *is relatively rare in developed countries [20], but in countries such as Africa, Thailand, and Brazil [3,5], it is a common cause of Gram-negative bacterial meningitis in infants with high mortality rates being reported. A study of infant *Salmonella *meningitis in Kuala Lumpur reported an 18% fatality rate with surviving infants experiencing considerable long-term neurological dysfunction (57%) and high relapse rates (38%) [3]. Although *Salmonella *meningitis is a rare complication of adult salmonellosis, the increased incidence of *Salmonella *bacteremia in HIV-infected patients [21,22], may expose such individuals to increased risk for more disseminated and protracted infections. Importantly, alarming increases in multi-drug resistance *Salmonella enterica *may be a harbinger for more virulent strains of *Salmonella *with increased invasive potential.

The ability of *S*. Typhimurium to infect the mouse brain was related to functional virulence loci including the type III secretion systems encoded in chromosomal genomic islands. In our studies over several years, infection of C57BL/6 mice with *S*. Typhimurium containing mutations in either of these secretion systems was never found to produce neurological deficits (over 1000 animals, data not shown) and mutant bacteria could not be recovered from the brains of representative infected mice (10 animals, data not shown). Neurological deficits were not unique to one particular strain of mouse, as we observed this behavior in five different mouse lines including wild type C57BL/6, wild type BALB/c, 129S1/SvImJ, 129S6-*Slc11a1tm1*^*Mcg*^, and B6.129-*Inpp5dtm1*^*Rkh*^. Much work has investigated the genetic factors in mice involved in resistance to *S*. Typhimurium infection [23]. These efforts have revealed a critical role for the *Slc11a1 *gene (formally Nramp1) in early innate resistance to *Salmonella *infection [17]. We note that 4 out of the 5 mouse strains used in our work carry a non-functional mutant allele of *Slc11a1 *(C57BL/6, BALB/c, 129S6-*Slc11a1tm1*^*Mcg*^, and B6.129-*Inpp5dtm1*^*Rkh*^). While we did observe neurological deficits in a mouse line that normally resists lethal infection due to a wild type *Slc11a1 *allele (129S1/SvImJ), our current sample size does not permit detailed conclusions to be drawn regarding the impact of *Slc11a1 *status on meningitis development. Thus, it is possible that both bacterial and host genetic factors are involved in infection of the brain and the development of meningitis. Further work using larger sample sizes and controls could address these issues. We cannot exclude hemiparesis in mice with neurological deficits and the potential involvement in this behaviour of the vestibular system will require additional investigation.

The ability of *Salmonella *to disseminate naturally to the brain in several mouse lines allows the use of the vast array of transgenic and knockout mouse strains to address the host genetic factors influencing CNS dissemination including the mode of bacterial transport from intestinal sites to systemic sites of infection, the role of various immune cells in the trafficking of intracellular salmonellae to the CNS and brain, and the role of various immune cell migration factors in this process. We suggest that the mouse model of *Salmonella *typhoid could represent a useful tool in which to investigate basic mechanisms of CNS infiltration and brain infection following oral ingestion of *Salmonella*. This model could also be exploited to examine the efficacy of investigational medicines (vaccines, anti-infectives) to treat invasive and protracted *Salmonella *infections. Importantly, this model fills a gap in the literature to address meningitis caused by naturally disseminating salmonellae.

## Conclusion

Oral infection of mice with *Salmonella enterica *serovar Typhimurium might represent a useful model in which to study the dissemination of a pathogen from the natural route of infection to the brain. Infection of the brain is followed by meningitis and a neurological deficit in a proportion of infected animals. Because more virulent strains of *Salmonella *are commonly associated with antibiotic resistance, the continued global epidemic spread of multi-drug resistant *Salmonella *isolates in human and animal medicine (such as *S*. Typhimurium DT104) presents a serious public health issue. Drug resistance in such isolates challenges our ability to treat life-threatening cases of salmonellosis, especially in infants and children. Oral infection of mice with *S*. Typhimurium is an accessible model in which to study the host and bacterial determinants that lead to dissemination and progression of infection from the gastrointestinal tract to the brain. We propose that this model has utility for testing new antibacterial chemotherapies to treat complicated, life-threatening *Salmonella *infections.

## Competing interests

The author(s) declare that they have no competing interests.

## Authors' contributions

MEW, JP, and BKC designed research, MEW, NFB, JP, and BKC performed research, MEW, JP, and BKC analyzed data, MEW, NFB, JP, BBF, and BKC wrote the paper. All authors read and approved the final manuscript.

## Pre-publication history

The pre-publication history for this paper can be accessed here:



## Supplementary Material

Additional File 1 Supplementary video 1. Movie showing rolling behavior of a C57BL/6 mouse infected with *Salmonella enterica *serovar Typhimurium at day 9 post-infection. Click here for file
